# Reduction in serum clusterin is a potential therapeutic biomarker in patients with castration-resistant prostate cancer treated with custirsen

**DOI:** 10.1002/cam4.93

**Published:** 2013-05-28

**Authors:** Brent Blumenstein, Fred Saad, Sebastien Hotte, Kim N Chi, Bernhard Eigl, Martin Gleave, Cindy Jacobs

**Affiliations:** 1 Trial Architecture ConsultingWashington, District of Columbia; 2 CRCUM-Université de MontréalMontréal, Quebec; 3 Juravinski Cancer CentreHamilton, Ontario; 4 BC Cancer Agency – Vancouver Cancer CentreVancouver, British Columbia; 5 The Vancouver Prostate CentreVancouver, British Columbia; 6 OncoGenex Pharmaceuticals Inc.Bothell, Washington

**Keywords:** Antisense oligonucleotide, chemotherapy, clusterin, custirsen, mCRPC

## Abstract

Elevated levels of clusterin (CLU), a stress-induced and secreted cytoprotective chaperone, are associated with advanced tumor stage, metastasis, treatment resistance, and adverse outcome in several cancers. Custirsen, a second-generation antisense oligonucleotide, inhibits CLU production in tumor cells and reduces serum CLU levels. A Phase 2 study evaluated custirsen in combination with second-line chemotherapy in men with metastatic castration-resistant prostate cancer (mCRPC) who had progressed while on or within 6 months of first-line docetaxel-based chemotherapy. Exploratory analyses evaluated serum CLU levels during custirsen treatment and correlative clinical effects on prostate-specific antigen (PSA) response, overall survival, and any relationship between serum CLU and PSA. Men with mCRPC were treated with mitoxantrone/prednisone/custirsen (MPC,* n* = 22) or docetaxel retreatment/prednisone/custirsen (DPC plus DPC-Assigned, *n* = 45) in an open-label, multicenter study. Subject-specific profiles of PSA and serum CLU levels during treatment were characterized using statistical modeling to compute subject-specific summary measures; these measures were analyzed for relationship to survival using proportional hazard regression. Estimated individual serum CLU response profiles were scored as below or at/above the median level for the population through 100 days postrandomization. Median survival was longer for subjects scoring below the median serum CLU level compared with subjects at/above the median level, respectively (MPC: 15.1 months vs. 6.2 months; DPC-Pooled: 17.0 months vs. 12.1 months). Lowered serum CLU levels during custirsen treatment when in combination with either chemotherapy regimen were predictive of longer survival in mCRPC. These results support further evaluation of serum CLU as a therapeutic biomarker.

Aside from PSA, there are currently no other prognostic or predictive biomarkers that can be used to guide treatment response in metastatic castration resistant prostate cancer (mCRPC). In a Phase 2 study, men with mCRPC were treated with prednisone and custirsen plus either mitoxantrone or docetaxel retreatment. Statistical modeling was used to compute subject-specific summary measures of PSA and serum clusterin levels at baseline and at Day 100 of treatment, followed by a regression analysis to evaluate relationship to overall survival. In this analysis, reduced serum clusterin levels during treatment were predictive of longer survival. These results currently support further evaluation of serum clusterin as a therapeutic biomarker in three ongoing Phase 3 clinical trials.

## Introduction

Clusterin (CLU) functions to protect cells from many varied therapeutic stressors that induce apoptosis, including androgen or estrogen withdrawal, radiation, and cytotoxic chemotherapy 1–4. CLU expression is regulated by HSF1 (also YB-1, EGR-1) and functions like small heat shock proteins (Hsps) to chaperone and stabilize conformations of proteins at times of cell stress 5. As a functional homologue of small Hsps, CLU has chaperone activity with a potent ability to inhibit stress-induced protein aggregation 6–7. CLU interacts with stressed cell-surface proteins (e.g., receptors) to inhibit pro-apoptotic signal transduction. CLU inhibits endoplasmic reticular (ER) stress by retro-translocating from the ER to the cytosol to inhibit aggregation of intracellular proteins and prevent apoptosis 8.

CLU also suppresses p53-activating stress signals and stabilizes the cytosolic Ku70-Bax protein complex to inhibit Bax activation 9. CLU specifically interacts with conformationally-altered Bax to inhibit apoptosis in response to chemotherapeutic drugs 10. In addition, CLU increases Akt phosphorylation levels and cell survival rates 11. CLU induces epithelial-mesenchymal transformation by increasing Smad2/3 stability and enhancing TGF-β-mediated Smad transcriptional activity 12. CLU also promotes prostate cancer cell survival by increasing NF-κB nuclear transactivation, acting as a ubiquitin-binding protein that enhances COMMD1 and I-kB proteasomal degradation via interaction with E3 ligase family members 13.

**Figure 1:**
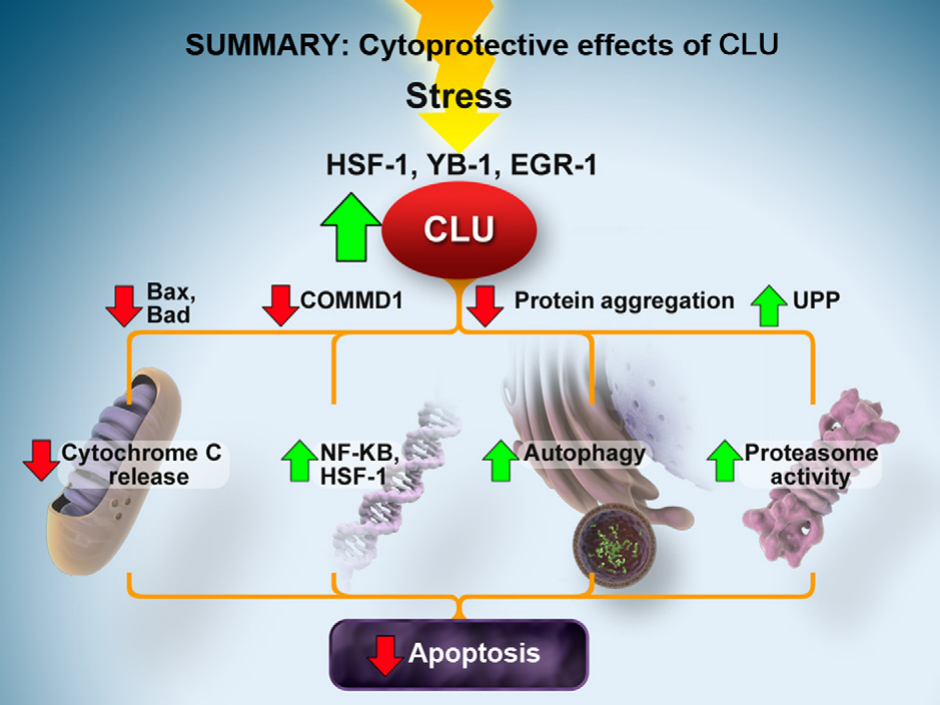
Role of CLU in cancer cell survival.

In preclinical models, CLU confers treatment resistance 14,15, while CLU inhibition potentiates activity of anti-cancer therapies 3,17. CLU expression has been correlated with higher serum prostate-specific antigen (PSA), higher clinical stage, metastatic disease, and shorter recurrence free and overall survival in prostate, bladder, and non-small cell lung (NSCL) cancers 1–23. An overall schema illustrating the role of CLU in cancer cell survival is shown in Figure 1.

Custirsen (OGX-011, TV-1011) is a second-generation antisense oligonucleotide (ASO) with affinity for CLU mRNA. Administration of custirsen has been shown to reduce CLU production within tumor cells and to lower serum CLU levels 22–26. Reduced CLU has been associated with enhanced treatment response in murine and human in vitro and in vivo models 18–31. In previous prostate cancer clinical studies evaluating the effects of custirsen treatment, tumor cell CLU levels were reduced by >94% and serum CLU levels were reduced by ~30% 22,25.

In a previously reported randomized Phase 2 study of subjects with metastatic castration-resistant prostate cancer (mCPRC), administration of custirsen with first-line docetaxel significantly reduced serum CLU levels within the first treatment cycle compared with docetaxel only 26. A survival benefit was also observed with custirsen plus docetaxel (median survival 23.8 months; 95% CI, 16.2 months-not reached) compared with docetaxel only (median survival 16.9 months; 95% CI, 12.8–25.8 months).

The initial objective of this study was to evaluate the feasibility of adding custirsen to either mitoxantrone/prednisone (MPC) or docetaxel/prednisone retreatment (DPC) as second-line chemotherapy in subjects with mCRPC who had disease progression within 6 months of completing first-line docetaxel-based chemotherapy. Results for the randomized study (42 subjects treated with custirsen and chemotherapy) have been previously published 22. Twenty-five additional subjects were treated with DPC under a protocol amendment, resulting in 67 total subjects randomized or assigned to custirsen treatment who received custirsen and chemotherapy. The objective of the statistical analyses reported herein was to evaluate the role of serum CLU at baseline and during treatment on overall survival. PSA assessments were also of interest in order to evaluate any relationship between changes in serum CLU and PSA during treatment and as a reference for assessing the relationship between serum CLU and survival.

## Material and Methods

### Study design

This was an open-label, randomized study of custirsen combined with second-line chemotherapy (docetaxel retreatment or mitoxantrone) in subjects with mCRPC. Based on a review of safety and efficacy data, the study was amended upon completion of randomization to allow additional enrollment into the docetaxel retreatment arm. The study was conducted at 10 Canadian sites. Study design and methods have been previously described 22. The study and amendment were reviewed and approved by all sites and Research Ethics Boards, and all subjects provided written informed consent.

Study treatment involved three loading doses of custirsen (OncoGenex Technologies, Inc., Vancouver, BC, Canada), 640 mg, administered on separate days as a 2-hour IV infusion during a 9-day period, followed by weekly administration on Day 1, 8, and 15 of each 21-day cycle. Prophylactic premedications included ibuprofen or acetaminophen. Subjects received prednisone (5 mg) orally twice daily for the treatment period unless intolerant. Chemotherapy consisted of either docetaxel (75 mg/m^2^ IV, 60 min) or mitoxantrone (12 mg/m^2^ IV, 30 min) administered on Day 1 of 21-day cycles. Up to nine cycles were permitted.

Serum CLU was assessed in samples collected at baseline, Day 1 of each cycle, and end of study treatment. Samples were analyzed utilizing solid-phase ELISA in microplate format (BioVendor Clusterin ELISA kit; Laboratorni medicina a.s., Czech Republic) designed for quantitative measurement of human CLU. Mayo Clinical Trial Services (Rochester, MN) validated and performed all serum CLU assays. PSA levels were assessed at clinical sites at baseline, Day 1 of each cycle, end of treatment visit, and during off-treatment follow-up. Overall survival (OS) was defined as the time from start of study treatment to death; OS was censored at the date of last follow-up for subjects still alive.

### Statistical analyses

Analyses focused on evaluation of chemotherapy regimen (mitoxantrone vs. docetaxel), serial PSA and serum CLU assessments, and survival. PSA and CLU were defined as biomarkers. Each subject's serial biomarker measurements through 1 year were fit to a quadratic model, and the estimated model in its functional form was used as a source of subject-specific summary measures or features 32. This method of feature extraction blunts the influence of subject-specific measurement variability and also enables use of data from subjects with missing baseline assessments. A Day 100 interval was chosen for estimating subjects' biomarker features as this represents approximately the time to complete four cycles of chemotherapy. The Day 100 interval puts the focus on early biomarker changes in response to study intervention and reduces interference from events preventing further biomarker assessments. PSA was analyzed as the base 10 logarithm of PSA, which is typically used in statistical models that evaluate PSA because the distribution of PSA has an extended right tail 33. For both PSA and CLU, subjects were excluded from analysis only if they had fewer than four assessments or if assessments ended before 21 days following initiation of study treatment; these exclusions were required by the statistical modeling used and are considered too few in number to induce significant bias.

For each biomarker and subject, baseline and follow-up values were computed from the estimated model for that subject. The biomarker baseline value was computed from the function at time zero. The Day 100 follow-up biomarker result from treatment, henceforth simply called the result for each biomarker, was the maximum or minimum value from the function at time zero through Day 100. If the quadratic function had a critical value (first derivative equal zero) in the interval 0 to 100, then the result was computed from the function at the time of this critical value; otherwise, the estimated follow-up level was computed from the model at Day 100. These computed baseline and result levels from each subject and biomarker were used to compute additional subject measures, such as change from baseline. In some cases, features were dichotomized for analytic purposes using a rounded value near the median.

Extracted features were analyzed for relationships to outcomes using graphics, descriptive statistics, Kaplan–Meier estimation, and proportional hazard regression. In this exploratory study, *P* values are used as indicators of model term inclusion and do not have meaning for assessing probabilistic hypothesis testing. A step-down hierarchical procedure was used in order to eliminate noncontributory terms in the models being explored, with criterion for inclusion of a model term being *P* < 0.1.

## Results

### Subjects

Forty-five subjects were randomized and 42 received treatment with custirsen in combination with chemotherapy, designated as MPC and DPC. Based on a review of safety and efficacy data in the randomized study, the original protocol was amended to enroll an additional 25 subjects (designated DPC-Assigned) who received the same intervention as the DPC group. When the DPC and DPC-Assigned groups are pooled, the resulting group is designated DPC-Pooled. The criteria for exclusion of individual subjects induced by using the quadratic modeling methodology resulted in the loss of 4/67 subjects, leaving 63 analyzable subjects.

### Extraction of profile features

**Figure 2:**
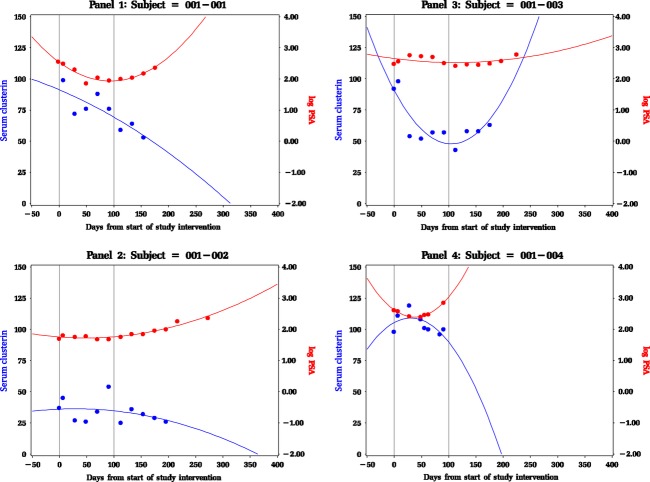
Serial serum CLU and log PSA values by time. Shown is a sample from the graphs created to assess the general shape of the individual longitudinal outcomes. These graphs also show the fit of the quadratic model to each individual's data.

Figure 2 shows measured serial CLU assessments and log PSA values by time for the first 4 of 63 subjects; these subjects illustrate the variety observed in the individual subject profiles and the quadratic model that fits to these subjects. Three subjects did not have measured baseline serum CLU assessments; the quadratic model was used to estimate a time = 0 value for these subjects. The median time to last serum CLU assessment within the first year for the 63 analyzable subjects was 156 days (range, 44–327 days; interquartile range [IQR], 94–197 days). The median time to last PSA assessment within the first year was 176 days (range, 44–365 days; IQR, 112–252 days).

**Figure 3:**
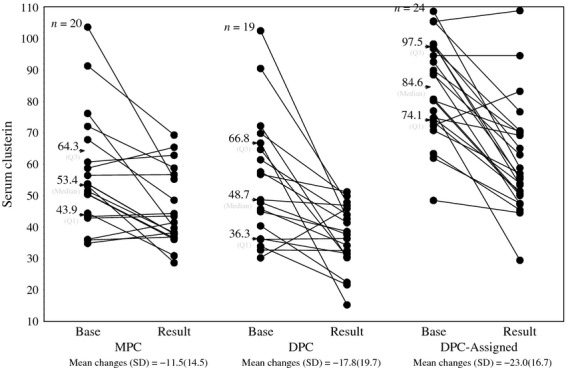
Serum CLU features and mean change by treatment group. For each group the extracted features of the 100-day CLU profile are plotted. These graphs show the general trend in response to the implementation of intervention.

**Figure 4:**
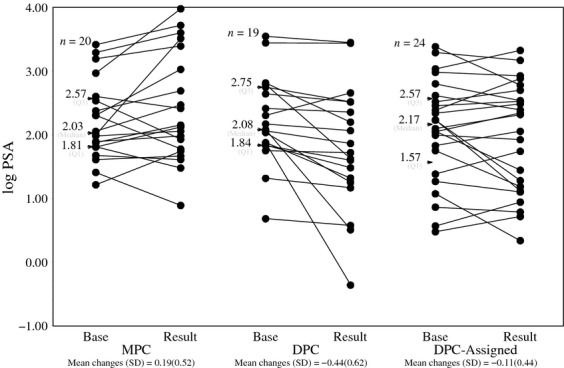
Log PSA features and mean change by treatment group. For each group the extracted features of the 100-day log(PSA) profile are plotted. These graphs show the general trend in response to the implementation of intervention.

Figures 3 and 4 show CLU and log PSA features for each subject's quadratic model, with lines connecting subject-specific biomarker baseline and Day 100 results. Median serum CLU baseline and Day 100 results were 64.7 μg/mL and 46.9 μg/mL, respectively; cutpoints of 64 and 47 are close to values in previous studies 22–23. Group-specific median log PSA at baseline and Day 100 were 2.08 ng/mL and 2.06 ng/mL, respectively, leading to cutpoints of 2. Cutpoints were subsequently used to partition subjects at baseline and Day 100 into low and high groups.

Estimated mean change values for CLU and log PSA are also shown in Figures 3 and 4. Estimates of mean changes from baseline to Day 100 did not exhibit a notable difference between groups. For subsequent analyses, DPC and DPC-Assigned groups were pooled.

### Subject characteristics and disposition

Baseline demographics at study entry did not differ materially between the three groups except for baseline CLU in the DPC-Assigned group, which was notably higher (Table 1).

**Table:** Baseline and Day 100 descriptive statistics for 63 subjects analyzed

Item	MPC	DPC	DPC-Assigned
Age			
*N*	20	19	24
Mean	63.8	67.1	64.6
Median	61	67	62.5
IQR	56.5–72.5	62–76	59.0–71.5
Range	49.0–81.0	48–80	53.0–80.0
Baseline PSA level			
*N*	20	19	24
Mean	478.5	515.0	394.6
Median	105.5	119.2	120.5
IQR	62.0–371.9	67.3–469.0	38.4–384.2
Range	16.8–2630.2	4.8–3570.8	3.1–2405.2
Baseline log PSA			
*N*	20	19	24
Mean	2.22	2.22	2.07
Median	2.03	2.08	2.17
IQR	1.81–2.57	1.84–2.75	1.57–2.57
Range	1.21–3.42	0.68–3.55	0.48–3.39
Estimated log PSA result			
*N*	20	19	24
Mean	2.42	1.79	1.96
Median	2.14	1.72	2.19
IQR	1.76–3.21	1.25–2.52	1.15–2.62
Range	0.89–3.98	−0.36 to 3.45	0.34–3.33
Baseline CLU			
*N*	20	19	24
Mean	56.9	54.7	84.4
Median	53.4	48.7	84.6
IQR	43.9–64.3	36.3–66.8	74.1–97.5
Range	34.8–103.7	10.2–102.6	48.5–108.8
Estimated CLU result			
*N*	20	19	24
Mean	45.4	38.9	61.4
Median	40.6	37.6	56.6
IQR	37.3–56.0	30.5–45.9	50.4–70.2
Range	28.6–69.3	15.2–51.3	29.4–109.0

The median number of treatment cycles was 6 in the MPC group and 7 in the DPC-Pooled group. Twenty-three of 67 subjects (34%) completed all 9 cycles. The percentage of subjects discontinuing treatment was similar for the MPC (64%) and DPC-Pooled (67%) groups. The main reason subjects discontinued treatment was progressive disease (42%). Additional reasons for discontinuation were adverse event (9%), withdrawal of consent (8%), physician decision (3%), and symptomatic disease progression (3%). No deaths occurred prior to Day 100.

### Effect of custirsen on serum CLU levels and PSA response

Changes in serum CLU results from baseline to Day 100 are shown by group in Figure 3. Mean changes are −11.5, −17.8, and −23.0 μg/mL for MPC, DPC, and DPC-Assigned groups, respectively; there is little evidence of between-group differences in these changes (*P* = 0.0944, one-way ANOVA). Across all three groups, 51/63 (81.0%) subjects had decreases; the overall mean change was −17.8 μg/mL and was significantly different from zero (*P* < 0.001) despite large standard deviations. Thus, the data as analyzed indicate that initiation of study intervention resulted in CLU decreases in most subjects.

Results in log PSA from baseline to Day 100 are shown by group in Figure 4. There is no evidence of substantial change in any group (mean changes +0.19, −0.44, and −0.11 ng/mL for MPC, DPC, and DPC-Assigned groups, respectively, with large standard deviations). Across all three groups, the mean change was −0.11 and was not significantly different from zero (*P* = 0.127).

There was no evidence of correlation between change in log PSA result and change in serum CLU result (data not shown). The Pearson correlation coefficient between change in log PSA and change in CLU was −0.065 (*P* = 0.61).

### Overall survival

Subjects were followed for a minimum of 36 months after the first dose of custirsen. Of 63 subjects analyzed in the statistical models, 57 had died.

**Figure 5:**
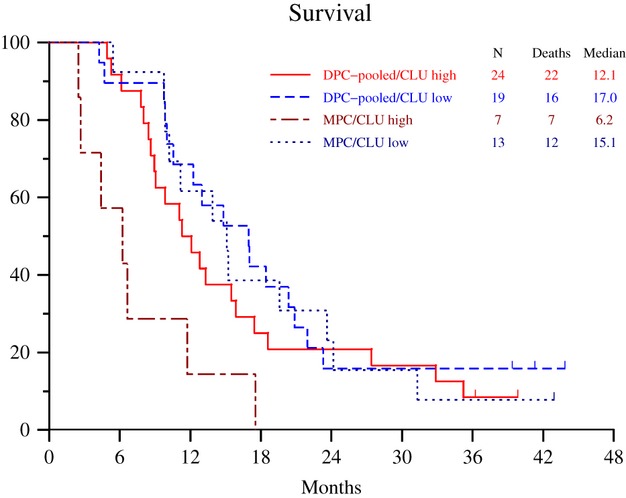
Kaplan–Meier estimates of survival by treatment group. The proportional hazard regression modeling (Table 2) suggested that Kaplan–Meier estimates from the four subgroups shown are a good representation of the data. In particular note that the difference between high versus low CLU response applies regardless of the type of chemotherapy.

Kaplan–Meier estimates of survival are shown in Figure 5 for the MPC and DPC-Pooled groups and Day 100 CLU results (low vs. high, defined with cutpoint of 47 μg/mL). In both chemotherapy groups, median survival was longer among subjects with a low Day 100 CLU result compared with subjects with a high CLU result. In the MPC group, median survival was 15.1 months for subjects with a low CLU result versus 6.2 months for subjects with a high CLU result. Similarly, in the DPC-Pooled group, median survival was 17.0 months for subjects with a low CLU result, versus 12.1 months for subjects with a high CLU result.

Proportional hazard regression modeling for survival was performed based on the following explanatory indicator variables: chemotherapy, baseline low PSA, baseline low CLU, decrease in PSA result, and low Day 100 CLU result. The starting model included all five explanatory variables and all possible interactions to provide a complete description of the data.

Baseline CLU level did not contribute to survival outcome in this model either as an explanatory variable or in any interaction. There was also no contribution or interaction between the log PSA and the low Day 100 CLU result variables in the model. Estimates from the model are shown in Table 2. Because the best fit model has no baseline CLU variable and there are no interactions between log PSA and the low Day 100 CLU result variables, the relationship between the low CLU result and survival can be illustrated without taking PSA into account at either baseline or posttherapy.

**Table:** Proportional hazard regression modeling for survival

Variable^1^,^2^	df	Parameter estimate	Standard error	Chi-square	Pr > Chi sq
Chemotherapy	1	−3.13848	0.70529	19.8019	<.0001
(0 = mitoxantrone vs. 1 = docetaxel)					
Low baseline CLU	Not contributory					
(0 = CLU > 64 vs. 1 = CLU ≤ 64)
Low baseline log PSA	1	−1.70813	0.66505	6.5967	0.0102
(0 = log PSA > 2 vs. 1 = log PSA ≤ 2)					
Low CLU result	1	−2.47114	0.70122	12.4190	0.0004
(0 = CLU > 47 vs. 1 = CLU ≤ 47)					
PSA favorable change	1	−3.87441	0.91230	18.0360	<.0001
(0 = no decrease vs. 1 = decrease)					
Interaction between chemotherapy and low baseline log PSA	1	3.02546	0.96097	9.9121	0.0016
Interaction between chemotherapy and PSA favorable change	1	2.87465	1.00921	8.1136	0.0044
Interaction between chemotherapy and low CLU result	1	2.54501	0.79178	10.3318	0.0013
Interaction between low baseline PSA and PSA favorable change	1	3.87054	1.16147	11.1051	0.0009
Two-way interactions including baseline CLU	All not contributory					
Two-way interactions: Low baseline log PSA by low baseline CLU and low log PSA result by low baseline CLU	Neither contributory					
Three-way interaction: chemotherapy by low baseline PSA by PSA favorable change	1	−5.83264	1.44222	16.3556	<.0001
Three-way interactions including any CLU variable	All not contributory					
Four-way interactions	All not contributory					
Five-way interaction	Not contributory					

1 Biomarker variables are based on feature extraction from the individual patient quadratic models.

2 The starting model included the five explanatory variables (i.e., chemotherapy regimen, baseline CLU, baseline log PSA, low CLU result, PSA favorable change) plus all possible interactions. The model used a step-down hierarchical procedure with the criterion for conclusion being a two-sided *P* < 0.1. Shown is the final model from the selection procedure.

The model does include the two-way interaction between chemotherapy and the low posttherapy CLU result variable. Chemotherapy-specific hazard ratio estimates (95% CI) are 0.272 (0.105–0.706) for MPC and 0.742 (0.389–1.416) for DPC-Pooled. These chemotherapy-specific hazard ratio estimates are less than unity, which suggests that the low CLU result variable is prognostic of improved survival specific to chemotherapy regimen. This prognostic relationship of chemotherapy-specific low Day 100 CLU results variable can be seen in the Kaplan–Meier estimates of survival shown in Figure 5.

## Discussion

The fact that survival was longer among subjects with a low serum CLU result during treatment compared with subjects with a high CLU result is consistent with preclinical data linking CLU expression to inhibition of treatment-induced cell death and with retrospective analyses of factors predictive for survival. Increased CLU levels have been reported in several cancers, including prostate, ovarian, and bladder and have been associated with poor prognostic features 20–35. In prostate cancer, Miyake et al. 36 showed that both serum CLU level and density (serum CLU level/prostate volume) were significantly higher in patients with prostate cancer compared with patients with benign prostatic hyperplasia. In addition, higher serum CLU levels in patients with prostate cancer were significantly associated with major prognostic factors such as high pretreatment serum PSA levels, advanced clinical stage, metastatic disease, and high percent of positive biopsy cores. Their findings showed that biochemical recurrence-free survival was significantly shorter in patients who had elevated CLU density at the time of radical prostatectomy compared with similar patients with normal CLU density.

Yang et al. 34 showed that CLU appeared to be both a biomarker associated with ovarian cancer and a prognostic factor associated with shorter survival. Overexpression of CLU in ovarian cancer tissue samples was found more often in advanced stage disease (*P* = 0.0001) compared with earlier disease. In addition, average survival time in patients with CLU overexpression was significantly shorter than in those with normal CLU expression (*P* = 0.033).

In patients with transitional cell carcinoma (TCC) of the bladder, Hazzaa et al. 21 observed that serum CLU levels were significantly higher in patients with TCC (invasive and superficial) compared with nontumor controls. The sensitivity and specificity of serum CLU as a tumor marker for TCC of the bladder were 80% and 91%, respectively. Furthermore, they showed that mean CLU mRNA expression in invasive disease tissue was significantly higher than in superficial disease or normal tissue 21. In addition, CLU expression levels correlated significantly with tumor grade and multiplicity but not with tumor size. They speculated that increased CLU expression was involved in TCC tumorigenesis and progression, especially since the recurrence-free survival time of patients with CLU overexpression was significantly shorter than that of patients with CLU underexpression (9.8 months vs. 35.2 months, respectively).

Another small retrospective study in patients with nonmuscle-invasive bladder cancer found a statistically significant association between bladder tissue CLU expression, measured by IHC, and progression to muscle-invasive disease following initial transurethral resection of the bladder tumor (TURBT) 37. The study authors suggest that tissue CLU in TURBT specimens may help identify patients who might benefit from more aggressive treatment earlier in disease.

Treatment with custirsen has been previously reported to reduce CLU levels and to be associated with longer survival outcomes in mCRPC and non-small cell lung cancer (NSCLC) 22–26. In a randomized, Phase 2 study in 82 patients with mCRPC evaluating docetaxel/prednisone as first-line chemotherapy with and without custirsen treatment, serum CLU levels were evaluated during the first cycle in all patients 26. Custirsen treatment significantly reduced serum CLU levels by a mean decrease of 26% by the end of cycle 1, compared to a slight increase of 0.9% in patients treated with only docetaxel/prednisone. Results from this study showed that, although there was no difference in tumor response rate, median survival for patients treated with custirsen plus docetaxel/prednisone was 23.8 months compared to 16.9 months for patients treated with docetaxel/prednisone only. In a single-arm, Phase 2 study of 81 patients with NSCLC evaluating custirsen with a first-line gemcitabine plus platinum regimen, 95% of patients had reductions in serum CLU from baseline by cycle 3. Mean reduction from baseline was 25 μg/mL (*P* < 0.0001; paired *t*-test for baseline vs. minimum CLU during treatment); median minimum serum CLU level was 38 μg/mL 23. Patients who had serum CLU levels ≤38 μg/mL during study treatment had a median survival of 27.1 months compared to 16.1 months for patients with serum CLU levels >38 μg/mL (*P* = 0.02).

Previously published results for the 42 randomized patients on this study showed that custirsen treatment significantly decreased serum CLU levels, with possible correlations between low serum CLU levels post treatment and prolonged survival 22. The additional analyses described herein were performed on all 63 analyzable subjects and characterized changes in each subject's Day 100 serum CLU and log PSA result following custirsen treatment, with assessment of the relationship of these changes to survival outcome. Low serum CLU results during treatment appeared to be highly predictive of improved survival outcome. In contrast, no relationship between PSA results and survival or between PSA results and serum CLU results was evident in this dataset. In other trial analyses, using different methods, a correlation between PSA decline and survival has been shown 38.

Our analysis was limited by not accounting for other baseline prognostic factors; thus, there may be confounding factors (e.g., overall disease burden, performance status) leading to bias. Although our analysis showed that baseline serum CLU levels were not prognostic for survival, the study design may have confounded these results; reduction in serum CLU levels due to custirsen treatment being administered to all subjects could have interfered with detection of a true baseline prognostic effect. In addition, one could further speculate that treatment stress in a control arm could result in increased CLU levels above baseline 26, and that increased CLU levels due to chemotherapy stress could be associated with treatment resistance or worse outcome. Ongoing randomized Phase 3 studies evaluating chemotherapy with and without custirsen will provide data on the prognostic value of baseline serum CLU as well as other possible confounding baseline factors.

The statistical modeling of the type of data analyzed is unavoidably difficult because multiple other outcomes are being related to the survival outcome. This is in contrast to models where only baseline attributes are used to predict survival. The potential biases inherent in relating multiple outcomes should be appreciated 39. As is typical of general statistical modeling efforts, many choices must be defined in the process of creating a model and some of these choices are driven by the data. The objective is to create a model that describes the data well enough to allow relationships between variables to be reliably assessed. In the words of George E. P. Box 40, “Essentially all models are wrong, but some are useful.” The main choices made for the modeling described herein included: minimum number of four serial biomarker assessments and assessments had to go beyond 20 days; quadratic shape of patient longitudinal profiles (ascertained to be adequate by viewing each patient profile); and 100 day cutoff for feature extraction (based on completion of four chemotherapy cycles, maximizing inclusion while defining a landmark for avoiding bias of early deaths given no deaths occurred before Day 100). These choices only disqualified four of the 67 subjects.

In summary, increased CLU levels have been previously correlated with various clinical and pathological parameters in a variety of tumors both as a possible prognostic biomarker and as a therapeutic biomarker during custirsen treatment. The ability to monitor serum CLU levels during custirsen treatment, whether combined with chemotherapy or with other cancer therapies (e.g., hormone or radiation), might allow individual assessments for potential survival benefit. The potential for serum CLU levels to serve as a therapeutic biomarker for custirsen treatment is being evaluated in ongoing Phase 3 studies enrolling over 2700 patients (clinical trial registration numbers: NCT01188187, NCT01578655, and NCT01630733). Prospective analyses from these Phase 3 trials will assess the validity of monitoring serum CLU level as a therapeutic biomarker.
